# Changes in perceived scientific consensus shift beliefs about climate change and GM food safety

**DOI:** 10.1371/journal.pone.0200295

**Published:** 2018-07-06

**Authors:** John R. Kerr, Marc Stewart Wilson

**Affiliations:** School of Psychology, Victoria University of Wellington, Wellington, New Zealand; Coventry University, UNITED KINGDOM

## Abstract

Despite an overwhelming scientific consensus, a sizable minority of people doubt that human activity is causing climate change. Communicating the existence of a scientific consensus has been suggested as a way to correct individuals’ misperceptions about human-caused climate change and other scientific issues, though empirical support is mixed. We report an experiment in which psychology students were presented with consensus information about two issues, and subsequently reported their perception of the level of consensus and extent of their endorsement of those issues. We find that messages about scientific consensus on the reality of anthropogenic climate change and the safety of genetically modified food shift perceptions of scientific consensus. Using mediation models we also show that, for both these issues, high consensus messages also increase reported personal agreement with the scientific consensus, mediated by changes in perceptions of a scientific consensus. This confirms the role of perceived consensus in informing personal beliefs about climate change, though results indicate the impact of single, one-off messages may be limited.

## Introduction

There are some issues for which a substantial proportion of the public hold beliefs that are at odds with broad scientific consensus. Examples include the reality of human-caused climate change [1,2], the safety of genetically modified food (GM) [3] and the efficacy of vaccines [4]. This ‘rejection of science’ poses a risk to humanity: doubts about climate change lead to personal and societal inaction, resulting in global environmental changes as well as social and economic costs [5]; concerns regarding vaccination threaten to lower immunisation rates and increase the spread of diseases such as measles [4], and; fears around the safety of GM foods could limit efforts to provide a sustainable level of food production for a growing global population [6].

To varying degrees, public disagreement over these issues exists in New Zealand. A 2014 representative national survey reported that less than half (49%) of New Zealanders feel certain that climate change is really happening (24% are undecided and 28% disagree) [7]. Thirty percent of participants in the 2014 wave of the longitudinal New Zealand Attitudes and Values study disagreed or were unsure that humans are causing climate change [2]. This disagreement and uncertainty is at odds with the scientific consensus that climate change is occurring and caused by humans. A recent review of studies examining the level of consensus estimates that 97% of climate scientists agree that climate change is happening and human-caused [8]. On the topic of GM, the most recent public opinion research suggests that New Zealanders’ tend to hold negative attitudes about GM foods and a plurality believe that they are dangerous to human health. In a 2013 random postal survey of New Zealanders, 27% of respondents were ‘totally opposed’ to GM and 42% agreed with the statement: ‘GM poses a significant risk to the health and safety of humans’ [9]. This is despite a number of comprehensive reports finding that GM foods are no different to conventional foods in terms of negative health effects [10,11]. Public opinion data on vaccination is sparse in New Zealand, but a 2012 nationally representative survey of adults reported that 90% of respondents thought that vaccines were safe. Of the remaining 10%, half the respondents had concerns about vaccine safety and half were unsure [12].

Along with personal beliefs regarding these issues, people also hold widely varying views on the level of consensus among scientists. For example, Leining and White’s [7] survey found that only 53% of New Zealand respondents agreed with the statement ‘Most scientists agree that humans are causing climate change.’ Misperceptions of scientific consensus have been attributed a range of overlapping factors, including: media giving equal balance to both sides of a debate [13], deliberate misinformation campaigns by interested parties [14] and cognitive biases arising from political and cultural ideologies [15]. Messages specifically communicating the scientific consensus (‘consensus messages’) have been suggested as a way to correct such misperceptions as well as shift individuals’ personal attitudes about scientific issues [14,16].

Consensus messages tap into two heuristics–mental rules-of-thumb–studied in persuasion research: the *expert heuristic* (‘experts can be trusted’) and the *consensus heuristic* (‘consensus implies correctness’). Within models of persuasion such as the Heuristic Systematic Model [17] and the Elaboration Likelihood Model [18], heuristics are employed, consciously or subconsciously, to reduce cognitive effort when evaluating a message. Information attributed to an expert (vs. non-expert) source has a greater influence on an individuals’ attitudes and opinions. Likewise, information that a position is supported by the majority of a group generally has more influence on attitudes than information stating a position is supported by a minority [19]. A number of studies examining the influence of consensus messages on beliefs draw on the heuristic role of expert consensus information as an explanation for its potential effects on individuals’ beliefs about scientific issues (e.g. [20–23]).

There are a growing number of experimental studies investigating the efficacy of consensus messages in changing perceptions and beliefs. These have primarily focussed on climate change but also touch on other scientific domains.

### Changing perceptions of scientific consensus and personal beliefs

#### Climate change

Cross-sectional studies typically show a positive relationship between the perception that there is widespread agreement among climate scientists and personal belief that climate change is occurring [23–25]. A number of experimental studies have found that exposing individuals to a simple message incorporating the ‘97%’ figure in various forms increases participant perceptions of scientific consensus, most commonly operationalised as an estimate of the percentage of scientists agreeing that climate change is occurring [26–29]. Studies have also shown that perception of consensus among climate scientists can be decreased by reading information from sceptic organisations claiming there is a lack of consensus [30], reading news reports giving disproportionate balance to sceptical viewpoints [31] or by watching a sceptical ‘climate conspiracy’ video [32]. Taken together, this research shows that individuals’ perceptions of a scientific consensus regarding climate change can be manipulated by relatively simple interventions.

Taking the next logical step, social psychologists have examined the possibility that changing people’s perception of the scientific consensus leads to changes in their personal belief that human-caused climate change is occurring. In an experiment, van der Linden and colleagues [22] show that reading messages about the 97% consensus significantly increases individuals’ estimates of scientific agreement on the reality of human-caused climate change. They use a path modelling approach to argue that these changes are, in turn, associated with increases in belief in human-caused climate change, concern over climate change and support for public action. The authors present their results as evidence supporting a ‘Gateway Belief Model’ (GBM), with perceptions of consensus acting as a key cognition influencing other beliefs. They write:

…we find that increasing public perceptions of the scientific consensus is significantly and causally associated with an increase in the belief that climate change is happening, human-caused and a worrisome threat. In turn, changes in these key beliefs are predictive of increased support for public action. In short, we find that perceived scientific agreement is an important gateway belief, ultimately influencing public responses to climate change. (p.1)

Similar experiments have demonstrated that informing individuals about the high level of scientific consensus significantly increases reported personal belief that human-caused climate change is occurring [27,33,34]. Conversely, several studies have reported that exposure to a consensus message may increase perceptions of scientific consensus, but has no effect on belief in the reality of climate change [28,35–38].

#### Genetically modified food

Like climate change, GM food safety is a topic of intense public debate [39,40], however, only one study to date has examined the role of consensus information in changing beliefs. Applying the GBM to the topic of genetic modification, Dixon [41] reports in two studies that exposure to a simple message stating “90% of scientists believe that genetically modified foods are safe to consume” increases participants’ perceptions of a scientific consensus on the issue, but, at a broad level, has no impact on their personal beliefs about GM food safety. Examining the data further, Dixon reports moderated mediation models describing the effect of a consensus message on beliefs mediated via perceptions of consensus. Here he reports a significant indirect effect of consensus message on beliefs, mediated via perceptions of consensus. This indirect effect was moderated by prior beliefs, such that the consensus message had a stronger effect on beliefs among participants with more positive prior attitudes towards GM food, and a weaker (in one study non-significant) effect for those with negative prior attitudes towards GM foods.

#### Other issues

While the majority of attention to consensus messaging has focused on climate change, studies have also explored other scientific issues. In support of the GBM, studies have found that interventions which change participants’ perception of a scientific consensus also change their personal beliefs in relation to vaccination [20,42,43], pharmaceutical pollution of waterways [44], scientific whale research and the link between blood type and personality [38].

#### Current study

At the heart of the GBM is the *causal* link between perceptions of scientific consensus and personal beliefs. While consensus information can shift perceptions of consensus, the absence of a subsequent effect on personal beliefs in some studies [35,36,41] calls into question this causal link, and the overall efficacy of consensus messaging as an approach to changing public opinion on climate change [45,46]. Further studies examining the role of perceived consensus across different scientific issues can add to this body of research, offering empirical insights building towards a clearer understanding of the role of consensus information in informing beliefs about scientific issues. Indeed, a recent report from the National Academy of Sciences has called for more research into how, and when, consensus information can shift individuals’ opinions, as well as comparison of communication approaches across differing national contexts [47].

Here we present a basic test of the GBM in a New Zealand student (convenience) sample, experimentally investigating the impact of simple consensus messages on self-reported personal beliefs, and the mediating role of perceptions of scientific consensus. The study examines two separate scientific issues where a scientific consensus exists but there is public uncertainty: climate change and GM food safety. Climate change was selected as it is the issue on which the GBM was initially based, and there are a number of conflicting findings in the literature (e.g., [36,38]). GM food safety was selected as there is only one study to date examining the GBM in this context [41]. While a number of studies have used consensus messages using a specific high percentage value to describe the level of consensus e.g. ‘97% of climate scientists agree…’, no studies investigating climate change or GM food beliefs have examined the effect of a similar message outlining a relatively low percentage. Does low consensus information have an identical but opposite effect on beliefs? In the current study, we include both high (97%) and low (63%) consensus messages to answer this question.

Based on research reviewed above, we expect that messages detailing a high or low level of agreement on GM food safety and climate change will respectively increase or decrease (compared to controls) participants’ perceptions of scientific consensus on these issues. Drawing on the GBM, we also expect that these messages will have an effect on participants’ beliefs about climate change and GM food safety. We predict that high consensus messages will increase participant’s agreement with the consensus position–that climate change is happening or that GM food is safe to eat–relative to a control group who read no message. Similarly, we predict that low consensus messages will decrease these beliefs. Lastly, we also predict that any effects of consensus messages on beliefs (specifically belief in human-caused climate change or belief that GM food is safe) can be explained by changes in perceptions of consensus. This hypothesis will be examined using statistical mediation models [48].

## Method

### Participants

A total of 696 participants took part in the study: 509 females (73.1%), 179 males (25.7%) and eight individuals reporting non-binary or no sex (1.2%). Participants were enrolled in an introductory psychology course at Victoria University Wellington and voluntary completion of the study counted as partial course credit towards required research participation/summarisation hours. Ages ranged from 17 to 52 years of age (*M* = 18.86, *SD* = 2.99). All participants provided informed consent via an electronic form and were fully debriefed at the conclusion of the study. The study was approved by the Victoria University of Wellington School of Psychology ethics committee (application number: 0000023961).

### Materials

Participants completed an online survey which contained an experimental manipulation. Participants read one of several consensus messages, or an attention check in the control condition (3 participants failing this check were removed). In the consensus message conditions, participants were presented with a single simple statement relating a high (97%) or low (63%) level of consensus regarding the reality of climate change or the safety of GM food. The climate change consensus messages read: *“Did you know*? *A recent survey shows that [97%][63%] of climate scientists agree that climate change is occurring and caused by humans*.*”* The GM messages were: “*Did you know*? *A recent survey shows that [97%][63%] of food scientists agree that genetically modified food is safe to eat*.*”* These statements were modelled on previous studies, which found that short simple statements significantly influence participants’ estimates of consensus [28,29,41]. The ‘high’ value of 97% consensus was selected as it reflects current estimates of scientific consensus regarding climate change [8]. Previous data from this student population indicate the mean estimate of scientific consensus among climate scientists to be approximately 80% [49] thus the figure of 63% was selected as the ‘low’ figure, being equidistant from this mean. As there were no prior data available for estimates of the level of scientific consensus on GM food safety in this population, the 97% and 63% values were used for consistency in an exploratory capacity. Participants who read a consensus statement also completed an attention check on the same page as the statement, entering a number in response to the following request: *To make sure you understood the text*, *could you please enter the percentage of scientists in agreement according to the statement above*. Participants who failed this check were removed from the sample (11 participants).

Both before and after the experimental manipulation, all participants were asked to provide an estimate of the percentage of climate scientists agreeing “that climate change is happening and caused by humans” and the percentage of food scientists agreeing “that food made from genetically modified plants and animals is safe to eat.” Responses were captured on a sliding scale ranging from 0% to 100%, with the marker anchored at 50%. Pre-treatment items provided a randomisation check as well as a baseline to ensure that the experimental manipulation did change perceptions of consensus. Following the experimental manipulation, participants completed a filler section comprising a shortened version of the National Science Foundation Science Literacy Scale [50], to increase the separation between the experimental manipulation and items capturing post-treatment consensus estimates and outcome variables [51]. Post-treatment consensus estimate items were embedded in a battery of six items asking participants to estimate the percentage of scientists agreeing with a range of statements that reflect debated science. These extra items were included to increase the psychological separation between the experimental manipulation and post-treatment GM/climate consensus items [51].

Lewandowsky, Gignac and Oberauer’s [52] 5-item scale was used to measure participants’ belief that human-caused climate change is occurring (example item: *Human CO2 emissions cause climate change*). Responses were captured on a 7-point Likert scale ranging from 1 (*Strongly disagree*) to 7 (*Strongly agree*). The scale displayed acceptable internal consistency (Cronbach’s *α* = .72) according to the criteria suggested by George and Mallery [53]. For comparison, Lewandowsky et al., report an *α* value of .78 for this scale.

Dixon’s [41] 3-item scale was used to measure belief that GM food is safe to eat (example item: *GM foods are safe to eat*). Responses were captured on a 7-point Likert scale ranging from 1 (*Strongly disagree*) to 7 (*Strongly agree*). The scale displayed good reliability (*α* = .85), slightly lower than that reported by Dixon (*α* = .91).

The GBM extends beyond simple alignment of beliefs with scientific consensus; research also suggests that shifting perceptions of consensus influences concern, support for policy and behavioural intentions [22,43]. A number of other scales covering these constructs were included in the survey, presented after the measures above, but are not reported here for brevity. Details of these scales and comparisons between experimental groups can be found in S2 File.

### Procedure

Participants registered to undertake the survey using a SONA Systems (Bethesda, MD) interface and completed the survey online via the SurveyMonkey platform (www.surveymonkey.com, San Mateo, CA) in their own time over a seven-day period. Participants were randomised to one of five conditions: reading a statement about a high or low level of scientific consensus regarding climate change or GM food, or a control condition. All participants completed all measures (a full list of all survey items can be found in S1 File). Data were analysed using SPSS v23.0 and mediation was tested using Hayes’ PROCESS macro for SPSS [54].

## Results

### Effects of consensus messaging on perceptions of consensus

#### Climate change

**Fig 1:**
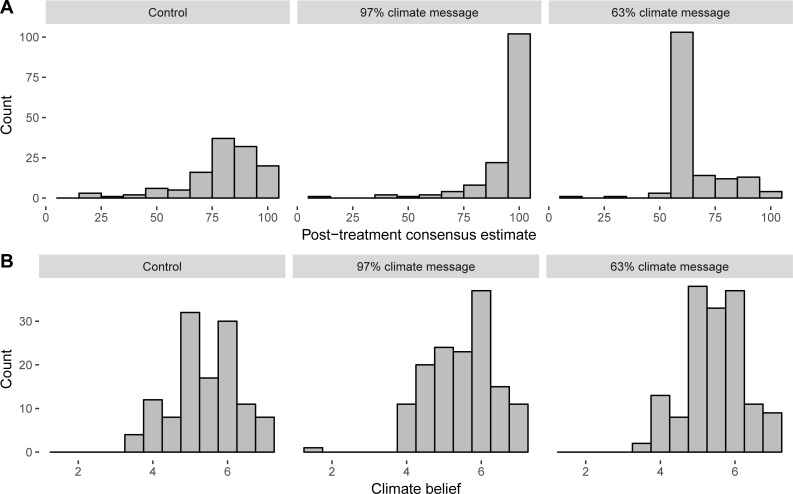
(A) Distribution of post-treatment estimates of scientific consensus on climate change and (B) average score on climate change belief scale in experimental conditions.

Mean estimates of consensus before and after reading a message about high (97%) or low (63%) consensus among climate scientists, or no message, are shown in Table 1. The distribution of consensus estimates and scores on the climate change belief scale, by condition, are shown in Fig 1.

**Table 1:** Mean estimates of consensus among climate scientists (SD) before and after reading climate change consensus messages.

	N	Pre-treatment estimate		Post-treatment estimate	
High (97%) climate message ^a^	142	79.35	(15.75)	92.34^bc^*	(11.97)
Low (63%) climate message ^b^	151	78.89	(17.27)	67.49^ac^*	(11.74)
Control ^c^	122	79.61	(15.24)	80.17^ab^	(16.87)

*Significant difference between pre- and post-treatment estimates, *p* < .001. Superscripts denote significant differences between conditions at each time point, *p* < .001.

We examined the effects of climate change consensus messages on estimates of agreement among scientists. A 2 (time: pre- vs post-treatment) x 3 (condition: high, low vs control messages) mixed measures ANOVA found a significant main effect of condition *F*(2, 412) = 36.80, *p* < .001, η_p_^2^ = .15. Post-hoc tests with Bonferroni correction indicated that the mean estimates of consensus (pre- and post-treatment combined) for all conditions were significantly different from each other (*p* < .001 in all cases), with estimates in the high consensus condition higher than those in the control condition and those in low consensus condition lower than the control condition. While there was no main effect of time, *F*(1,412) = 0.84, *p* > .1, there was a significant interaction between time and message condition, *F*(2,412) = 87.04, *p* < .001. η_p_^2^ = 0.30. We followed up the significant interaction with post-hoc tests. As expected, there was no significant difference between pre- and post-treatment estimates of consensus in the control condition *t*(121) = 0.51, *p* > .1. In the high consensus message condition, post-treatment estimates of consensus were higher than pre-treatment estimates *t*(141) = 8.74, *p* < .001, *d* = .92. In the low consensus message condition, post-treatment estimates of consensus were lower than pre-treatment estimates, *t*(150) = 8.44, *p* < .001, *d* = .92. Univariate analyses indicated no significant difference across conditions in pre-treatment estimates, *F*(2,412) = 0.07, *p* > .1, and a significant difference between groups for post-treatment estimates *F*(2,412) = 123.81, *p* < .001, η_p_^2^ = .37. Comparisons of post-treatment estimates with Bonferroni correction revealed that estimates in the high consensus message condition were higher than those in the low and control conditions and estimates in the low condition were lower than those in the control condition (*p* < .001 in all cases). In sum, reading messages about high or low consensus among climate scientists, respectively, increases or decreases perceptions of consensus.

#### GM food safety

**Fig 2:**
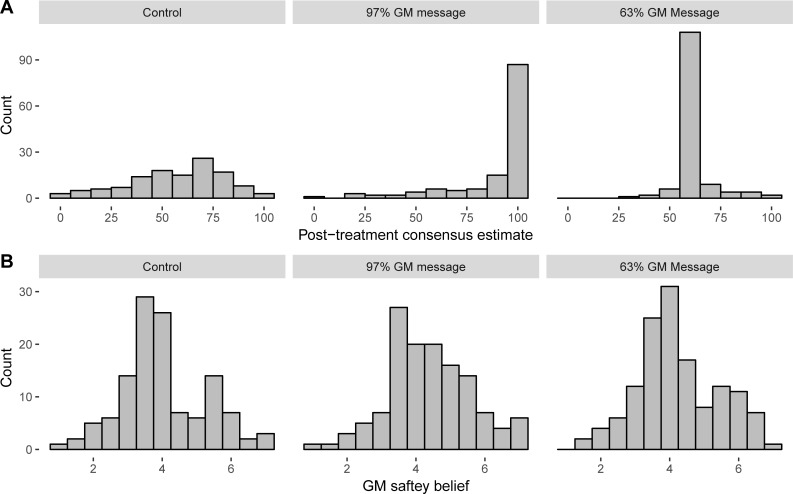
(A) Distribution of post-treatment estimates of scientific consensus on GM food safety and (B) average score on GM food safety beliefs scale in experimental conditions.

Mean estimates of consensus before and after reading a message about high (97%) or low (63%) GM consensus among food scientists, or no message, are shown in Table 2. The distribution of consensus estimates and scores on the GM food safety beliefs scale, by condition, are shown in Fig 2.

**Table 2:** Mean estimates of consensus among food scientists (SD) before and after reading GM consensus messages.

	*N*	Pre-treatment estimate		Post-treatment estimate	
High (97%) GM message ^a^	131	56.95 ^b^	(24.03)	87.30^bc^*	(19.39)
Low (63%) GM message ^b^	136	63.95^a^	(24.33)	64.25^ac^	(8.70)
Control ^c^	122	57.31	(22.07)	57.56^ab^	(23.16)

*Significant difference between pre- and post-treatment estimates, *p* < .001.

Superscripts denote significant differences between conditions at each time point at *p* < 0.05.

A 2 (time: pre- vs post-treatment) x 3 (condition: high, low vs control messages) mixed measures ANOVA showed a significant main effect of message condition *F*(2, 386) = 22.06, *p* < .001, η_p_^2^ = .10. Post-hoc tests with Bonferroni correction indicated that the mean estimates of consensus in the high consensus message group were higher than those in the low message (*p* < .001) and control (*p* < .001) conditions. Mean consensus estimates in the low consensus message condition were also significantly higher than those in the control condition (*p* < .01). There was a significant main effect of time *F*(1,386) = 81.05, *p* < .001, η_p_^2^ = .17, indicating a significant difference between pre- and post-treatment scores across all groups combined. There was also a significant interaction between time and condition, *F*(2,386) = 77.29, *p* < .001, η_p_^2^ = .29. We followed up this interaction with paired-samples t-tests comparing pre- and post-treatment consensus estimates in each message condition separately. Post-treatment consensus estimates were higher than pre-treatment estimate in the high consensus message condition, *t*(130) = 12.58, *p* < .001, *d* = 1.38. There was no significant difference between pre- and post-treatment consensus estimates in the control condition, *t*(121) = 0.20, *p* > .1, or in the low consensus message condition *t*(135) = 0.15 *p* > .1. A one-way ANOVA indicated a significant effect of message condition for post-treatment consensus estimates, *F*(2, 386) = 97.40, *p* < .001, η_p_^2^ = .33. Post-hoc comparisons indicated that both the high (*p* < .001) and low (*p* < .01) consensus message groups had higher estimates of scientific consensus compared to the control group and were significantly different from each other (*p* < .001). A significant difference was observed between conditions for pre-treatment estimates, *F*(2, 386) = 3.72, *p* = .02, η_p_^2^ = .02. Post-hoc comparisons showed that pre-treatment estimates of the scientific consensus on GM food safety were significantly higher in the low consensus message condition compared to the high consensus message condition (*p* = .047). The difference between the low and control conditions was marginally significant (*p* = .073), with estimates in the low condition higher than those in the control condition. Overall these results indicate that reading a message about high scientific consensus on the safety of GM food among food scientists increases individuals’ perceptions of scientific agreement on the issue. Given that the ‘low’ GM consensus message described a level of consensus close to pre-treatment estimates and did not significantly alter participant’s perceptions of consensus relative to pre-treatment estimates, we remove this group from subsequent analyses (combining the no message group and the low GM consensus group into a single control group did not substantially alter results, see S3 File).

### Effects of consensus messages on beliefs

A one-way ANOVA indicated no significant differences in scores on the climate change beliefs scale between participants who read a high consensus climate message (*M* = 5.47, *SD* = 0.88), those who read a low consensus message (*M* = 5.44, *SD* = 0.80) and the control group (*M* = 5.44, *SD* = 0.80; *F*(2, 412) = 0.26, *p* > .1). For GM food safety beliefs, an independent samples t-test revealed that participants who read a high consensus message reported higher scores on the GM food safety beliefs scale (*M* = 4.37, *SD* = 1.12) than those who read a control message (*M* = 4.02, *SD* = 1.23; *t*(251) = -2.31, *p* = .02). This indicates that reading a message about high or low consensus among climate scientists had no impact on reported belief in human-caused climate change, but reading a message about a high level of consensus on the safety of GM food among food scientists increased self-reported personal belief that GM food is safe to eat.

#### Mediation analyses

We undertook mediation analyses to examine if consensus messages had an indirect effect on beliefs, mediated via perceptions of consensus. Mediation allows the investigation of the effect of an independent variable (*X*) on a dependent variable (*Y*) through a third ‘mediating’ variable (*M*), based on a series of regression analyses. The indirect effect *ab* is the product of the effect of *X* on *M* (*a*) and the effect of *M* on *Y* accounting for *X* (*b*). Any effect of *X* on *Y*, accounting for *M*, is termed the direct effect (*c’*) and the overall effect of *X* on *Y* is termed the total effect (*c*). Earlier guidelines for mediation from Baron and Kenny’s [55] ‘causal steps’ approach recommend only undertaking analyses to examine the role of a mediating variable when there is a significant total effect (i.e. a significant relationship between the independent and dependent variable; path *c*). Here we follow more recent guidelines which reject this approach and propose that mediation is a useful tool in identifying mediating relationships even in the absence of a total effect [56,57]. Each model presented below separately considers the effects of a message condition compared to the control group as the independent variable, coded dichotomously (message condition = 1, control = 0). Here we also control for pre-treatment estimates of consensus, thus we are measuring how post-treatment *changes* in perceived consensus relate to climate change belief. To our knowledge, this approach has not been employed in previous mediation models examining the role of perceived consensus in influencing beliefs (e.g. [29,58]). We note that van der Linden et al. [22] used pre-post score differences for all variables in their path model. The current study captured pre- and post-test consensus estimates (as a within-subjects manipulation check) but only post-test responses to the climate change and GM belief items.

#### Climate change

**Fig 3:**
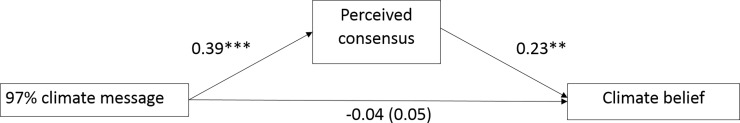
The effect of a 97% climate message on belief in human-caused climate change, mediated via perceptions of consensus among climate scientists, controlling for pre-treatment estimates. Standardized regression coefficients. **p < .01,***p < .001. *N* = 264.

A mediation model examining the effect of a 97% climate message on climate beliefs, via perceptions of consensus, found a significant effect of message on the mediator, which in turn was significantly associated with belief in human-caused climate change (Fig 3; full details of each mediation model are reported in S3 File).

The unstandardized indirect effect, *ab* = .16, 95% CI [.05, .27], was significant: the lower and upper values of a 95% bias-corrected bootstrap confidence interval (5000 samples) did not include zero [56]. Interpreted in practical terms, this means the shift in perceived consensus caused by reading a message about high scientific consensus is associated with a 0.16 increase in climate change endorsement captured on a 7-point scale. We also report here the partially standardised indirect effect, *ab*_*ps*_ which describes the indirect effect of a one unit change in *X* expressed in terms of the standard deviation of the outcome variable *Y*. As the independent variable in the mediation model is dichotomous, this approach provides greater ease of interpretation than the completely standardised indirect effect, *ab*_*cs*_ [59]. The partially standardised indirect effect, *ab*_*ps*_ = .19, 95% CI [.05, .32], implies that reported belief in human-caused climate change increases by approximately one-fifth of a standard deviation due to the indirect effect of reading a high consensus message, via changes in perceptions of consensus.

**Fig 4:**
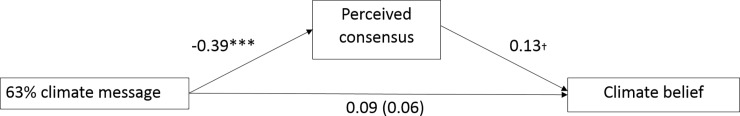
The effect of a 63% climate message on belief in human-caused climate change, mediated via perceptions of consensus among climate scientists, controlling for pre-treatment estimates. Standardized regression coefficients. †*p* < .10, ***p < 001. *N* = 273.

The mediation model for the low climate consensus condition is detailed in Fig 4. Reading a message about a low (63%) level of agreement among climate scientists did not have a significant indirect effect on belief that human-caused climate change is occurring, *ab* = -.09, 95% CI [-.20, .02]; *ab*_*ps*_ = -.11, 95% CI [-.26, .03]. This indicates that changes in estimated consensus caused by reading a low consensus message do not have a subsequent effect on belief that human-caused climate change is occurring.

#### GM food safety

**Fig 5:**
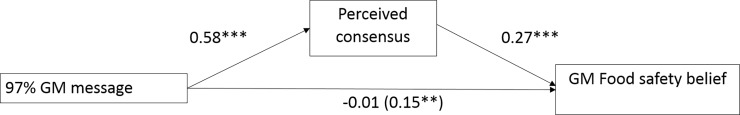
The effect of a 97% GM message on belief that GM food is safe to eat, mediated via perceptions of consensus among climate scientists, controlling for pre-treatment consensus estimates. Standardized regression coefficients. ***p < .001. *N* = 253.

Recall that, as the exploratory ‘low’ GM consensus message conveyed a figure close to the participants’ existing estimates of scientific, we chose not to further analyse the low consensus GM condition data. The mediation model for the high GM consensus condition is outlined in Fig 5. Reading a message about a high (97%) level of agreement among food scientists on the safety of GM food had a significant positive indirect effect on beliefs about the safety of GM food, *ab* = 0.39, 95% CI [0.18, 0.62]; *ab*_*ps*_ = 0.35, 95% CI [.17, .55]. Reading a high consensus message increased participants’ estimates of the scientific consensus, which in turn was associated with greater belief that GM food is safe. The partially standardised indirect effect indicates that the reading a high consensus GM message led to approximately a third of a standard deviation shift in GM food safety beliefs, mediated via perceptions of consensus.

In summary, for both the climate change and GM food issues, mediation models revealed an indirect effect of high consensus information on scientific beliefs in the expected direction. Reading messages about high consensus among scientists increases estimates of scientific consensus. Post-treatment estimates of consensus, controlling for the experimental manipulation and pre-treatment estimates of consensus, are significantly positively associated with personal beliefs aligning with the scientific consensus, supporting the GBM. Reading a message outlining a low level of scientific consensus (63%) did not have a significant total or indirect effect on belief that human-caused climate change is occurring.

## Discussion

This study offers new insights into the efficacy of consensus messaging and the role that perceptions of scientific agreement play in informing personal beliefs. The GBM posits that people’s beliefs about scientific issues are influenced by their perceptions of what scientists believe [22]. It follows that interventions which change an individual’s perception of a scientific consensus on a given issue would also change their beliefs about the issue. We used an experimental approach to investigate the effect of consensus messages on participants’ perceptions of a scientific consensus on, and beliefs relating to, climate change and the safety of GM food.

Our results indicate that short, simple messages about high scientific consensus increase individuals’ perception of a scientific consensus. The mean estimate of scientific agreement on climate change increased from approximately 79% to 92% for participants who read a high consensus message. This replicates results from previous studies [26–29,41,60]. In the case of climate change, we also observed a surprisingly symmetrical effect for messages about relatively low consensus (mean estimates decreasing from 79% to 67%). Quantitative information about the percentage of scientists endorsing the reality of climate change appears to increase or decrease perceptions of agreement in a roughly equal manner. Regardless of any subsequent effects on beliefs, the current results support the conclusion that messages about the scientific consensus are effective in correcting misperceptions about the actual level of scientific agreement on climate change. This is a worthwhile endpoint in itself, as highlighted in recent studies investigating the effects of climate sceptic communications seeking to decrease public perception of scientific consensus [30,31].

Despite the results showing that messages about scientific agreement on climate change significantly influence perceptions of consensus, we did not find that consensus messages had a *total* effect on climate change beliefs, i.e. we did not find a significant relationship between reading or not reading the high consensus climate message and belief in climate change. On its own, this result suggests that simple messages about the level of agreement among climate scientists have no influence on individuals’ belief in the reality of human-caused climate change. We should note that our between-subjects design (measuring climate change beliefs only after the experimental manipulation) has lower power to detect a direct effect of message condition on beliefs, compared to a within-subjects, pre-post design (see [22,34]). A further consideration is the possibility of a functional ceiling effect. Our control group appeared to have relatively high acceptance of climate change (discussed further below). While there was certainly capacity for higher scores to be recorded on the scale for most participants in the high consensus message condition (see Fig 1B), it is possible that few participants are willing to report greater agreement with all items in the climate change acceptance scale—even if they strongly believe that human-caused climate change is occurring. Although we do not report a main effect of message condition on climate beliefs, our mediation analyses found that a high climate consensus message did have a significant *indirect* effect; reading a message significantly influenced perceptions of consensus, which in turn influenced key beliefs. This mediated effect of high consensus messages has been noted in several previous experiments, most notably in the path analysis presented in the seminal GBM study [22] but also others [29,58]. Our mediation model examining low climate consensus message effects did not support the GBM, however. We find that reading a low consensus climate message decreased perceptions of consensus, but that this change did not have a negative effect on reported belief that human-caused climate change is occurring.

Previous studies which found no effect of consensus messages on climate change beliefs have only examined the total effect of message condition on beliefs and did not include perceived consensus as a mediator of this effect [35,36]. We note that mediation analyses may be better powered to detect an indirect effect compared to a total effect [57,61]. If we take a literal view of the GBM, then the absence of a total effect in our study might be taken as indicating that a simple, one-off message about consensus may not have in itself much practical value in changing beliefs. However, the results of the current study confirm the theoretical causal path outlined in the Gateway Belief Model: perceptions of consensus play a role in informing beliefs about climate change–at least for high consensus messages. More engaging or detailed interventions highlighting the scientific consensus on climate change (e.g. [58]) may hold greater promise as potential communication strategies. Likewise, repeated exposure to the ‘97%’ message could be expected to have a greater impact on beliefs [62,63]. Messages about the scientific consensus also compete with counterclaims that there is not a scientific consensus—often driven by vested interests [64,65]. Combining consensus messages with ‘inoculating’ warnings about incorrect counterclaims can prevent misinformation from neutralizing the effects of consensus messages[30,31]. At a national level, there is some evidence supporting the efficacy of consensus messaging. Hamilton [66] documents a growing awareness of the scientific consensus and acceptance of human-caused climate change in the US in recent years, “compatible with the proposition that implicitly or explicitly communicating evidence of agreement among scientists encourages public acceptance of [anthropogenic climate change] itself” (p. 9)

The mediating role of perceived consensus is apparent when considering messages about GM food safety. Here, the significant total effect of a high consensus message on GM food safety belief is explained by effects mediated via perceptions of consensus. This finding supports the generalisation of the GBM to scientific domains other than climate science, in agreement with van der Linden et al. [43], who found that consensus messages influenced beliefs about vaccine safety via perceptions of consensus. Future research should further examine the role of perceived scientific agreement in other science-related societal debates where public opinion diverges from scientific consensus, for example, community water fluoridation [67]. Our GM message results, to some extent, align with the only previous study examining consensus messaging in the context of GM food safety. Dixon [41] reported that a high consensus message had no overall effect on GM food safety belief (measured using the same scale as the current study). However, Dixon did report a significant indirect effect of a simple message on beliefs via perceptions of consensus (study 2). While we also report an indirect effect, we found a significant main effect of message condition on GM beliefs where Dixon does not. A potential explanation for the divergent findings is the nature of the consensus message; Dixon’s consensus message outlined that “90% of scientists believe GM foods are safe to consume,” while the current study used a higher figure of 97%. Future studies could compare the effects of several differing levels of high consensus (e.g. 90% vs 97%) on beliefs about GM foods (see [29,68,69]). In the context of genetically modified food, simple messages highlighting consensus may be an effective way of increasing public agreement with current consensus on GM food safety. Although we must acknowledge that the high consensus figure used in the GM message was selected to correspond with the climate change figure and is unlikely to reflect the current (and as-yet unquantified) level of scientific agreement on the safety of GM food. It is important to also note that the current study focused solely on the issue of GM food safety, for which there exists a reasonably strong scientific consensus [11]. The overarching issue of acceptance of genetically modified organisms rests on wider considerations, including environmental and economic impacts [70].

Several limitations of the study must be acknowledged. Firstly, this study was undertaken in student sample and the results may not be generalisable to the wider population. For example, females were over-represented in our sample. Student samples are also typically more liberal than the general population, and political ideology is a predictor of opinions on both climate change and genetic modification [3]. Although political ideology was not measured in our study, the average estimate of the scientific consensus on climate change in our control group (80%) was higher than that reported for online samples in the US and Australia (usually in the range of 50–70% [29–31]). Similarly, the average score on the climate change acceptance scale in our control group was higher than that reported by Lewandowsky et al. for a US online sample using the same items (but with a 5- instead of 7-point Likert scale [52]). A rough comparison can be drawn by converting averages to Percent of Maximum Possible (POMP) scores [71]: the average POMP score on the climate change belief scale for the control group in the current study was 74% while Lewandowsky et al. [52] report 54% in their sample. Thus we must caution the reader that the results of the current study are drawn from a sample which appears to have, on average, a higher level of climate change acceptance than seen in more representative samples.

Many of the consensus messaging studies reviewed above were undertaken using US online samples (e.g. Amazon’s Mechanical Turk). The current study using a New Zealand student sample shows that the core tenet of the GBM–the mediating role of perceived scientific consensus–holds true in populations relatively different from that in which it has been most often assessed. As noted above, our sample was more accepting of the reality of human-caused climate change than participants in a similar US study. Further research with more representative or targeted samples is required to confirm that the pattern of influence outlined in the GBM remains consistent in individuals with a higher degree of scepticism regarding anthropogenic climate change. Future research should also examine the effect of ‘low’ consensus messages about GM food safety. Efforts in the current study were hampered by a lack of pilot data, leading to a ‘low’ figure which conveyed a level of consensus close to the existing estimates of the sample (i.e. did not reduce perceptions of consensus as intended). Descriptions of both a scientific consensus and lack thereof on the issue of GM food safety can be found in the mainstream media (e.g. [72,73]). Examining the effects of such messages and how they influence beliefs will aid our understanding of how public opinion on GM food is shaped by such information.

More generally, further research into consensus messaging alone and in combination with other communications strategies, such as framing (e.g. [36]), is warranted. Such studies will contribute to building an evidence-informed toolbox of communication strategies for scientists, journalists, and policymakers seeking to engage the public on the challenge of anthropogenic climate change and other publicly-debated scientific issues.

## Supporting information

S1 File Scale items. (DOCX)

S2 File Effects of consensus messages on concern, intentions and policy opinions. (DOCX)

S3 File Full mediation models. (DOCX)
