# Supplementary material for: Changes in perceived scientific consensus shift beliefs about climate change and GM food safety
Source: PLoS One. 2018 Jul 6;13(7):e0200295. doi: 10.1371/journal.pone.0200295 (PMC6034897; doi:10.1371/journal.pone.0200295)
Supplement: S1 File — (DOCX) [file pone.0200295.s001.docx]

**Supplementary File 1: Scale items**

*reverse scored item.

| **Pre-/ post-treatment consensus estimates [0-100 sliding scale]** | What percentage of climate scientists do you think agree that climate change is happening and caused by humans?  What percentage of food scientists do you think agree that food made from genetically modified plants and animals is safe to eat? |
| --- | --- |
| **GM Food safety belief [7-point Likert scale]** | *Please rate your agreement with the following statements*  GM foods are safe to eat  GM food can be harmful to public health*  GM ingredients in food can cause illness* |
| **Climate belief [7-point Likert scale]** | The climate is always changing and what we are currently observing is just natural fluctuation.*  Most of the warming over the last 50 years is due to the increase in greenhouse gas concentrations.  The burning of fossil fuels over the last 50 years has caused serious damage to the planet’s climate.  Human CO2 emissions cause climate change.  Humans are too insignificant to have an appreciable impact on global temperature.* |
| **Climate concern [7-point scale; not at all concerned – very concerned]** | *Please indicate your level of concern in response to the questions below*  How concerned, if at all, are you about climate change?  Considering any potential effects of climate change which there might be on you personally, how concerned, if at all, are you about climate change?  Considering any potential effects of climate change there might be on society in general, how concerned are you about climate change? |
| **Climate policy support [5-point scale; strongly oppose - strongly support]** | *Below are some actions the New Zealand government could take in relation to climate change or GM food. Please let us know how much you would support or oppose these policies.*  Lowering GST and increasing tax on petrol by 23c per litre  Providing tax rebates for people who purchase electric vehicles  Including farmers in the Emissions Trading Scheme (requiring them to pay for greenhouse gas emissions from livestock and operations)  Subsidising the cost of insulating houses to make them more energy efficient |
| **Anti GM policy [5-point scale; strongly oppose - strongly support]** | Requiring all food sold in New Zealand containing ingredients from GM plants and animals to be labelled as such  Removing restrictions on growing GM crops in New Zealand*  Banning the sale of food containing GM ingredients in New Zealand  Requiring meat and other products from animals fed GM crops to be labelled as such |
| **Climate intentions [7-point Likert scale]** | *Please let us know how much do you agree or disagree with the following statements*  I intend to help reduce climate change by changing my behaviour  I intend to do my bit to help tackle climate change  I intend to address climate change by taking personal action |
| **GM intentions [7-point Likert scale]** | I plan to never eat genetically modified food  I will only buy ‘GM-free’ food products  I always check the label to see food contains genetically modified ingredients  I intend to never buy food containing GM ingredients |
