# Supplementary material for: Changes in perceived scientific consensus shift beliefs about climate change and GM food safety
Source: PLoS One. 2018 Jul 6;13(7):e0200295. doi: 10.1371/journal.pone.0200295 (PMC6034897; doi:10.1371/journal.pone.0200295)
Supplement: S2 File — (DOCX) [file pone.0200295.s002.docx]

**Supplementary File 2: Effects of consensus messages on concern, intentions and policy opinions**

The Gateway Belief Model also posits that increases in belief that human caused climate change is occurring have a ‘cascading’ effect on related attitudes and beliefs, increasing concern about climate change and support for public action. In the current study we included a number of attitudinal measures to investigate the wider effects of consensus messages. While not the focus of the study, for completeness we report here these measures and compare results between experimental groups. A full list of all survey items can be found in File S1.

**Measures**

***Climate concern:*** A 3-item scale from Spence, Poortinga and Pidgeon [1] was used to measure concern regarding the effects of climate change was measured using. These items covered the three main types of concern associated with climate change: general concern, personal concern and societal concern (example: *Considering any potential effects of climate change there might be on society in general, how concerned are you about climate change?*). Participants responded on a scale ranging from 1 (*Not at all concerned*) to 7 (*Very concerned*). The scale displayed good reliability (*α* = .88), similar to that reported in Spence et al. (*α* = .83).

***Climate intentions:*** Personal intentions to act to address climate change were measured using a 3-item scale developed by van der Linden [2]. Rather than focusing on specific actions, this scale captures an overall general intent (example: *I intend to do my bit to help tackle climate change*). Participants indicated their agreement with the statements on a Likert scale ranging from 1 (*Strongly disagree*) to 7 (*Strongly agree*). The scale displayed excellent reliability (*α* = .94), higher than that reported by van der Linden (*α* = .85),

***Climate policy support:*** An *ad hoc* policy support scale was constructed to measure participants’ support for climate mitigation policy options. The design was modelled on previous scales [3,e.g., ,4] but incorporated four real world, New Zealand-specific climate change mitigation policy options drawn from media reports and policy documents [5–7]. For these items and the GM policy items detailed below, participants were provided with the following instructions: *Below are some actions the New Zealand government could take in relation to climate change or GM food. Please let us know how much you would support or oppose these policies* (example item: *Providing tax rebates for people who purchase electric vehicles*). Participants indicated their level of support for each policy on a 5-point scale ranging from 1 (*strongly oppose*) to 5 (*strongly support*). The reliability of this scale was poor (*α* = .56), however Cronbach’s *α* tends to be artificially deflated when there are only a small number of items in a scale. The mean inter-item correlation was .25 and the corrected item-total correlations for all items were above .30 which, as a rule of thumb, indicates that the items are measuring the same construct and could be usefully retained [8].

***GM intentions:*** Participants’ personal intentions regarding GM food (eating, purchasing and checking labels) were measured using a 4-item scale adapted from Spence and Townsend [9]. Two additional items were added to the original 2-item scale to improve reliability (example item: *I intend to never buy food containing GM ingredients*). Responses were captured on a 7-point Likert scale. The scale displayed good reliability (*α* = .86).

***GM policy support:*** As with the climate policy support scale, GM policy preferences were measured using an *ad hoc* scale comprised of four New Zealand policy options sourced from the media and policy documents [10–13]. Most relevant policy discussion relating to GM food safety has focused on restrictions around GM food availability and labelling. As such, the wording of items in this scale means that higher values indicate greater support for restrictions and regulations for GM food (example: *Banning the sale of food containing GM ingredients in New Zealand*). Participants indicated their level of support for each policy on a 5-point scale ranging from 1 (*strongly oppose*) to 5 (*strongly support*).The scale displayed marginal acceptability (*α* = .68).All corrected item-total correlations were greater than .30 and the mean inter-item correlation was .35.

**Results**

A multivariate ANOVA found no significant differences between high and low consensus message and control conditions across all dependent climate variables, *F* (8, 818) = .871, *p* = .54; Wilk's *Λ* = 0.983, η_p_^2^ = .01 (table S2.1). That is, participants who read a message about high or low consensus regarding the reality of human-caused climate change did not significantly differ from the control group in terms of: belief in the reality of human-caused climate change, concern over climate change, intentions to personally act to mitigate climate change or support for climate change policies. This finding doesn’t support the Gateway Belief Model, which posits that interventions which change perceptions of consensus should also change personal beliefs about climate change.

**Table S2.1. Descriptive statistics for climate-related outcomes after reading a high or low climate consensus message or no message.**

|  | Climate belief | | Concern | | Policy support | | Intentions | |
| --- | --- | --- | --- | --- | --- | --- | --- | --- |
|  | *M* | *SD* | *M* | *SD* | *M* | *SD* | *M* | *SD* |
| High (97%) climate message | 5.47 | (0.88) | 5.78 | (1.18) | 3.64 | (0.62) | 5.09 | (1.05) |
| Low (63%) climate message | 5.44 | (0.80) | 5.65 | (1.06) | 3.54 | (0.58) | 4.97 | (1.05) |
| Control | 5.40 | (0.87) | 5.58 | (1.10) | 3.49 | (0.57) | 4.84 | (1.20) |

A MANOVA examining GM food safety beliefs, intentions and policy preferences indicated a significant difference between the high GM consensus message and control conditions, *F* (3, 249) = 3.12, *p* = .03; Hotelling’s *T^2^* = 5.73, η_p_^2^ = .04 (table S2.2). Independent samples t-tests revealed significant differences between the message and control conditions for GM food safety beliefs, *t*(251)=-2.31, *p* = .02, *d* = 0.29, and anti-GM policy support *t*(251)= 0.54, *p* = .008, *d* = 0.34. These results indicate that exposure to a message about a high level of scientific agreement regarding the safety of GM food increases individuals’ reported belief that GM food is safe and lowers their support for policies regulating GM food products. These findings offer support for the Gateway Belief Model which predicts that changes in perceptions of scientific consensus are causally related to changes in personal beliefs.

**Table S2.2. Descriptive statistics for GM-related outcomes after reading a high GM consensus message or no message.**

|  | GM food safety | | GM intentions | | Anti GM-policy | |
| --- | --- | --- | --- | --- | --- | --- |
|  | *M* | *SD* | *M* | *SD* | *M* | *SD* |
| High (97%) GM message ^a^ | 4.37 ^b^ | (1.21) | 3.02 | (1.24) | 3.31^b^ | (0.64) |
| Control ^b^ | 4.02 ^a^ | (1.23) | 3.11 | (1.25) | 3.51 ^a^ | (0.55) |

Superscripts denote significant difference at *p* < .05.

**Discussion**

The current study found that consensus messages had no overall effect on concern over climate change, personal intentions to act or support for climate mitigation policies, as seen in previous studies examining similar constructs [14–16]. Exceptions include an experiment by Bolsen et al. [17] which found that while a high consensus message did not influence belief in human-caused climate change, it did increase support for climate policies and willingness to take personal action. A large (N = 3,150) survey experiment undertaken by van der Linden et al. [18] also found that a single consensus statement led to significant within-subjects increases in worry about climate change and general support for action. Taken in the context of this previous research, the current results suggest that any immediate effects of consensus messaging on more distal beliefs related to climate change, such as personal intentions and policy support, are at best small and difficult to detect.

This is the first study to date to examine the effects of consensus messages on personal intentions to buy or consume GM food and GM policy preferences. The results imply that actors seeking to decrease support for GM regulation would find consensus messaging a useful tactic in shifting public opinion.

**References**

1. Spence A, Poortinga W, Pidgeon N. The psychological distance of climate change. Risk Anal. 2012;32: 957–972. doi:10.1111/j.1539-6924.2011.01695.x

2. van der Linden SL. The social-psychological determinants of climate change risk perceptions, intentions and behaviours: a national study. [Internet]. The London School of Economics and Political Science. 2014. Available: http://etheses.lse.ac.uk/896/

3. Leiserowitz A. Climate change risk perception and policy preferences: The role of affect, imagery, and values. Clim Change. 2006;77: 45–72. doi:10.1007/s10584-006-9059-9

4. Dietz T, Dan A, Shwom R. Support for climate change policy: Social psychological and social structural influences. Rural Sociol. 2007;72: 185–214. doi:10.1526/003601107781170026

5. Royal Society of New Zealand. Transition to a low-carbon economy for New Zealand [Internet]. Wellinton; 2016. Available: https://royalsociety.org.nz/assets/documents/Report-Transition-to-Low-Carbon-Economy-for-NZ.pdf

6. Parliamentary Commissioner for the Environment. Climate change and agriculture: Understanding the biological greenhouse gases [Internet]. Wellington, NZ; 2016. Available: http://www.pce.parliament.nz/media/1678/climate-change-and-agriculture-web.pdf

7. Polkinghorne J. Guest post: carbon taxes and the NZ economy - Greater Auckland [Internet]. 2016 [cited 23 Aug 2017]. Available: https://www.greaterauckland.org.nz/2016/11/17/guest-post-carbon-taxes-and-the-nz-economy/

8. Field A. Discovering statistics using SPSS. 3rd ed. London: SAGE Publications; 2009.

9. Spence A, Townsend E. Examining consumer behavior toward genetically modified (GM) food in Britain. Risk Anal. 2006;26: 657–670. doi:10.1111/j.1539-6924.2006.00777.x

10. Peters G, Heinemann J. Should NZ grow genetically modified crops? - NZ Herald. In: New Zealand Herald [Internet]. 2012 [cited 23 Aug 2017]. Available: http://www.nzherald.co.nz/business/news/article.cfm?c_id=3&objectid=10833317

11. Galloway J. NZ will have to allow GM food to keep up with other countries | Stuff.co.nz. In: NZ Farmer [Internet]. 2016 [cited 23 Aug 2017]. Available: http://www.stuff.co.nz/business/farming/78586826/nz-will-have-to-allow-gm-food-to-keep-up-with-other-countries

12. GE Free NZ. Proposal to stop labelling of GM food would deny consumers basic right to know. [Internet]. 2016 [cited 23 Aug 2017]. Available: http://press.gefree.org.nz/press/20160823.htm

13. NZ Parliament. Primary Production Committee report on Petition 2011/68 of Jon Muller [Internet]. Wellington, NZ; 2015. Available: https://www.parliament.nz/resource/en-nz/51DBSCH_SCR62256_1/081c26f143ac1696ec551b82bad4e1f004985779

14. van der Linden SL, Leiserowitz AA, Feinberg GD, Maibach EW. How to communicate the scientific consensus on climate change: plain facts, pie charts or metaphors? Clim Change. 2014;126: 255–262.

15. Deryugina T, Shurchkov O. The effect of information provision on public consensus about climate change. PLoS One. 2016;11. doi:10.1371/journal.pone.0151469

16. Bolsen T, Druckman JN. Do Partisanship and Politicization Undermine the Impact of Scientific Consensus on Climate Change Beliefs? First Annual Research Roundtable on Global Climate Change Governance: Geoengineering. Chicago, IL; 2017. Available: http://www.law.northwestern.edu/research-faculty/searlecenter/events/roundtable/documents/Druckman_Partisan_Group_Identity_Belief_in_Human-Caused_Climate_Change.pdf

17. Bolsen T, Leeper TJ, Shapiro MA. Doing what others do: Norms, science, and collective action on global warming. Am Polit Res. 2014;42: 65–89. doi:10.1177/1532673X13484173

18. van der Linden S, Leiserowitz A, Maibach E. Gateway illusion or cultural cognition confusion? J Sci Commun. SISSA; 2017;16: A04.
