# Supplementary material for: Changes in perceived scientific consensus shift beliefs about climate change and GM food safety
Source: PLoS One. 2018 Jul 6;13(7):e0200295. doi: 10.1371/journal.pone.0200295 (PMC6034897; doi:10.1371/journal.pone.0200295)
Supplement: S3 File — (DOCX) [file pone.0200295.s003.docx]

**Supplementary File 3: Mediation models.**

Tables S3.1-3 outline the regression models used to determine the beta values for the paths in Figs 1-3 in the main text.

The first model in each table represents the effect of reading a high consensus message on climate beliefs (*c’*; the total effect). The second model shows the effect of the message condition on perceptions of consensus (*a*) and the final model shows the effect of both perceptions of consensus and message condition on climate beliefs (b and c respectively).

These analyses include pre-treatment perceptions of consensus as a covariate in all paths, thus interpreting the mediator as change in perceived consensus [1].

**Table S3.1. Effect of 97% climate consensus message on climate beliefs mediated via perceptions of consensus, controlling for pre-treatment estimates of consensus.**

|  | Outcome | | | | | | | | |
| --- | --- | --- | --- | --- | --- | --- | --- | --- | --- |
|  | Climate change belief | | | Post treatment consensus estimate | | | Climate change belief | | |
|  | *B* | SE | *β* | *B* | SE | *β* | *B* | SE | *β* |
| Constant | 4.03*** | 0.28 |  | 44.86*** | 4.19 |  | 3.46 | 0.33 |  |
| Message (vs. control) | 0.08 | 0.10 | 0.05 | 12.29*** | 1.57 | 0.39*** | -0.08 | 0.11 | -0.04 |
| Pre-treatment consensus estimate | 0.02*** | 0.00 | 0.30*** | 0.44*** | 0.05 | 0.44*** | .01** | 0.00 | 0.20** |
| Post-treatment consensus estimate |  |  |  |  |  |  | .01** | 0.00 | 0.23** |
|  |  |  |  |  |  |  |  |  |  |
| *R^2^* | .09 |  |  | .34 |  |  | .13 |  |  |

Unstandardized and standardized regression coefficients shown. ***p* > .01****p* < .001.

**Table S3.2. Effect of 63% climate consensus message on climate beliefs mediated via perceptions of consensus, controlling for pre-treatment estimates of consensus.**

|  | Outcome | | | | | | | | |
| --- | --- | --- | --- | --- | --- | --- | --- | --- | --- |
|  | Climate change belief | | | Post treatment consensus estimate | | | Climate change belief | | |
|  | *B* | SE | *β* | *B* | SE | *β* | *B* | SE | *β* |
| Constant | 3.89*** | 0.24 |  | 42.42*** | 3.69 |  | 3.59*** | 0.29 |  |
| Message (vs. control) | 0.06 | 0.09 | 0.04 | -12.34*** | 1.46 | -0.39*** | 0.15 | 0.11 | 0.09 |
| Pre-treatment consensus estimate | 0.02*** | 0.00 | 0.37*** | 0.47*** | 0.04 | 0.50*** | 0.02*** | 0.00 | 0.31*** |
| Post-treatment consensus estimate |  |  |  |  |  |  | 0.01† | 0.00 | 0.13† |
|  |  |  |  |  |  |  |  |  |  |
| *R^2^* | .14 |  |  | .41 |  |  | .15 |  |  |

Unstandardized and standardized regression coefficients shown. †*p* < .10 , ****p* < 001.

**Table S3.3. Effect of 97% GM consensus message on GM food safety beliefs mediated via perceptions of consensus, controlling for pre-treatment estimates of consensus.**

|  | Outcome | | | | | | | | |
| --- | --- | --- | --- | --- | --- | --- | --- | --- | --- |
|  | GM food safety beliefs | | | Post-treatment consensus estimate | | | GM food safety beliefs | | |
|  | *B* | SE | *β* | *B* | SE | *β* | *B* | SE | *β* |
| Constant | 2.63*** | 0.20 |  | 31.01*** | 3.33 |  | 2.23*** | 0.22 |  |
| Message (vs. control) | 0.36** | 0.14 | 0.15** | 29.90*** | 2.32 | 0.58*** | -0.02 | 0.17 | -0.01 |
| Pre-treatment consensus estimate | 0.02*** | 0.00 | 0.45*** | .46*** | 0.05 | 0.41*** | 0.02*** | 0.00 | 0.34*** |
| Post-treatment consensus estimate |  |  |  |  |  |  | 0.01*** | 0.00 | 0.27*** |
|  |  |  |  |  |  |  |  |  |  |
| *R^2^* | .22 |  |  | .50 |  |  | .26 |  |  |

Unstandardized and standardized regression coefficients shown. ***p* > .01, ****p* < .001.

**Incorporating low consensus GM message into control group for GM analyses.**

The ‘low consensus’ (‘63%’) GM message used in this study was in fact close to the extant level of perceived consensus in our sample, and did not significantly affect consensus estimates. As such we ran supplementary analyses including this group in the control condition to increase statistical power. The results of the GM analyses were not substantively changed (table S4).

**Table S3.4. Effect of 97% GM consensus message on GM food safety beliefs mediated via perceptions of consensus, controlling for pre-treatment estimates of consensus (low GM consensus and no message conditions combined as control group).**

|  | Outcome | | | | | | | | |
| --- | --- | --- | --- | --- | --- | --- | --- | --- | --- |
|  | GM food safety beliefs | | | Post-treatment consensus estimate | | | GM food safety beliefs | | |
|  | *B* | SE | *β* | *B* | SE | *β* | *B* | SE | *β* |
| Constant | 2.62*** | 0.15 |  | 40.40*** | 2.35 |  | 2.06*** | 0.20 |  |
| Message (vs. control) | 0.35** | 0.11 | 0.14** | 27.52*** | 1.75 | 0.59*** | -0.03 | 0.14 | -0.01 |
| Pre-treatment consensus estimate | 0.02*** | 0.00 | 0.48*** | .34*** | 0.03 | 0.37*** | 0.01*** | 0.00 | 0.39*** |
| Post-treatment consensus estimate |  |  |  |  |  |  | 0.02*** | 0.00 | 0.25*** |
|  |  |  |  |  |  |  |  |  |  |
| *R^2^* | .24 |  |  | .45 |  |  | .28 |  |  |

Unstandardized and standardized regression coefficients shown. ***p* > .01, ****p* < .001.

In this model we report a significant indirect effect, *ab* = 0.38, 95% CI [0.21, 0.57]; *ab_ps_* = 0.35, 95% CI [.20, .52]. This is comparable to the results reported in the main text comparing the 97% GM message group to the control group only (*ab* = 0.39, 95% CI [0.18, 0.62]; *ab_ps_* = 0.35, 95% CI [.17, .55]), with slight reductions in the size of the confidence intervals.

**References**

1. Valente MJ, MacKinnon DP. Comparing Models of Change to Estimate the Mediated Effect in the Pretest–Posttest Control Group Design. Struct Equ Model A Multidiscip J. 2017;24: 428–450. doi:10.1080/10705511.2016.1274657
